# Myeloid Leukemias and Virally Induced Lymphomas in Miniature Inbred Swine: Development of a Large Animal Tumor Model

**DOI:** 10.3389/fgene.2015.00332

**Published:** 2015-11-20

**Authors:** Raimon Duran-Struuck, Abraham J. Matar, Christene A. Huang

**Affiliations:** ^1^Department of Pathobiology, University of Pennsylvania School of Veterinary Medicine, PhiladelphiaPA, USA; ^2^University of Central Florida College of Medicine, OrlandoFL, USA; ^3^Center for Transplantation Sciences, Massachusetts General Hospital, BostonMA, USA

**Keywords:** swine, CML, PTLD, model, cancer, transplantation immunology

## Abstract

The lack of a large animal transplantable tumor model has limited the study of novel therapeutic strategies for the treatment of liquid cancers. Swine as a species provide a natural option based on their similarities with humans and their already extensive use in biomedical research. Specifically, the Massachusetts General Hospital miniature swine herd retains unique genetic characteristics that facilitate the study of hematopoietic cell and solid organ transplantation. Spontaneously arising liquid cancers in these swine, specifically myeloid leukemias and B cell lymphomas, closely resemble human malignancies. The ability to establish aggressive tumor cell lines *in vitro* from these naturally occurring malignancies makes a transplantable tumor model a close reality. Here, we discuss our experience with myeloid and lymphoid tumors in major histocompatibility characterized miniature swine and future approaches regarding the development of a large animal transplantable tumor model.

## Introduction

Malignancies of the hemolymphatic system in swine were first reported as early as 1865(Bostock and Owen, 1973) but generally, there are limited reports describing neoplasias in swine. This is partly due to the fact that most swine die at a relatively young age, either slaughtered for food or used in biomedical research. As of yet, there is no large animal tumor model available that can be reliably induced and consistently reproduced. The vast majority of documented cases of leukemias and lymphomas in veterinary oncology are in the domestic population (Schiffman and Breen, 2015). Liquid neoplasias have been reported in farm animals; however, these are uncommon due to the lack of desire for a clinical workup and preference for euthanasia to minimize animal suffering. Here, we will discuss how swine provide an attractive large animal model for the study of cancer biology and its treatment. Specifically, the Massachusetts General Hospital (MGH) miniature swine herd retains unique genetic characteristics that facilitate the study of hematopoietic cell (HCT) and solid organ transplantation (SOT; Hanekamp et al., 2011). A significant incidence of spontaneous chronic myeloid leukemias and herpesvirus associated B cell lymphomas have been reported in the MGH miniature swine herd, making it a viable option for the development of a large animal tumor model (Hanekamp et al., 2011).

## Swine As A Large Aminal Research Model

The use of large animal models in biomedical research remains controversial. Ethical justifications and selection of a less expensive model must continuously be addressed to private, government, and academic reviewers. However, the use of large animal models is important as murine studies often fail to translate clinically (Hunter and Williams, 2002; Adelman et al., 2006). Among the available large animals in biomedical research, primate models have the obvious advantage of physical and physiological similarity to humans, but there are several barriers to their use including strict regulation standards, expense, negative societal impression, difficulty in breeding and handling, prolonged time to sexual maturity and potential for infectious disease. Canine models are a more practical option and have been widely used (Cain and Champlin, 1989; Ladiges et al., 1990; Storb et al., 2001; Zaucha et al., 2001). However, no canine tumor model exists yet, and compared to swine, their use is less favored because of their status as a common companion animal. Alternatively, swine are an ideal experimental model for several reasons including ease of breeding and handling, short gestation periods, large litters, short time to sexual maturity, and an anatomy and physiology that closely resembles that of humans (Laber et al., 2002; Swindle et al., 2012). However, as with any animal model, there are limitations to the swine model. The ability to consistently reproduce findings in outbred species, though clinically relevant, remains the biggest challenge in terms of developing a tumor model.

Although murine studies have historically been critical in the study of cancer biology and immunological diseases, attempts at extrapolation to large animals or clinical studies have often been unsuccessful, especially with respect to studies of transplantation (Bortin, 1970; van Bekkum, 1984; Storb, 2003). This can be appreciated in studies of immunological tolerance, in which numerous approaches to allograft tolerance have been developed in mice, but very few have proven successful in clinical studies (Storb, 2003). Over 30 years ago, NIH researcher Dr. David Sachs initiated a selective breeding program of miniature swine to develop and maintain a large animal model for studies of transplantation biology (Sachs et al., 1976). Through years of selective breeding, Sachs et al. (1976) were able to “fix” the major histocompatibility (MHC) genes of the miniature swine herd, while retaining variability to minor antigens (miHAs; Pennington et al., 1981a,b; Mezrich et al., 2003). The homozygosity of MHC genes has made the MGH miniature swine a valuable model in that different clinical transplant scenarios can be mimicked (full MHC match, complete MHC mismatch, haploidentical match, etc). One line of swine was selectively inbred, which will refer to as the “SLA^dd^” line (swine leukocyte antigen - dd), aiming to achieve complete syngeneity (Hanekamp et al., 2011), as has been done in mice. Currently, the SLA^dd^ line has reached a coefficient of inbreeding of >94%. Despite not yet being 100% genetically identical, skin and organ allografts transplanted between animals within this line are consistently accepted without any immunosuppression (Mezrich et al., 2003). Extrapolating from these data, spontaneous tumors arising in this line can be harvested expanded *in vitro* and cryopreserved for *in vivo* transfer studies, providing the foundation for a transplantable swine tumor model. Here, we discuss our experience with myeloid and lymphoid tumors within the MGH miniature swine and future goals of a large animal tumor model.

## Chronic Myelogenous Leukemia In Swine

Recently, we reported a significant incidence of spontaneous myeloid leukemias in the inbred SLA^dd^ line of the MGH miniature swine herd and demonstrated that swine chronic myelogenous leukemias (CML) closely resembled human CML (Duran-Struuck et al., 2010). In our study, two swine CML cell lines were karyotyped to assess the presence of a specific translocation or mutation, similarly to the t(9;22) translocation, or philadelphia chromosome (Ph+), which is well documented in the majority of human CMLs (Oettel et al., 1994; Marks et al., 2010; Chereda and Melo, 2015). In both cases, a shortened chromosome arm was identified (Duran-Struuck et al., 2010), reminiscent of the classical Ph+ in humans. PCR was performed using BCR–ABL gene-specific primers to determine whether the genetic change was identical (or similar) to the Ph+ chromosome in human CML (Chereda and Melo, 2015). Sequences surrounding the known chromosomal breakpoint of the BCR and ABL genes in the human K562 CML cell line were compared to the available porcine or bovine sequences to identify conserved regions for primer design. Two bands of 300 and 500 nucleotides were detected in the pig while a single distinct band of 450 nucleotides was present in the K562 sample. Due to differences in chromosome numbers between humans and swine (23 pairs for humans and 19 for swine), we could not directly translate the t(9;22) translocation observed in humans CMLs. Future genetic studies of these swine tumors may provide a platform for novel therapeutic approaches for human tumors sharing similar genetic defects.

## An Inducible Swine Cml Model

Central to the development of many murine tumor models has been the establishment of *in vitro* oncologic cell lines. Similarly, characterization of tumor cell lines derived from inbred miniature swine and adaptation for *in vivo* growth is a possibility. Several CML cell lines from the SLA^dd^ inbred line were previously isolated from affected animals (Cho et al., 2007; Duran-Struuck et al., 2010), and aggressive subclones were selected out by serial passages *in vitro. In vivo* growth of a CML cell line originating in animal 14736 was assessed after direct inoculation into naïve swine conditioned with gamma irradiation (ranging from 100 to 500 cGy). Subcutaneous (SQ) injection of the 14736 CML cell line into an animal conditioned with 300 cGy total body irradiation (TBI) resulted in SQ tumor growth, but not systemic growth (Cho et al., 2007). Systemic tumorigenesis (with mostly lung involvement) required at least 500 cGy of TBI. Though non-myeloablative, 500 cGy of TBI proved to be significantly immunosuppressive and animals often died of infections and not due to the induced neoplasm. If animals received less irradiation (<500 cGy), tumor cells did not grow *in vivo* (Duran-Struuck, unpublished data). Injection of CML cell lines directly into the bone marrow (intra-BM) of SLA^dd^ swine conditioned with low levels of TBI (100–200 cGy) also did not lead to systemic leukemic growth. BM biopsies from one animal that had been infused with intra-BM CML were cultured *in vitro.* The resulting cell line was phenotypically and morphologically similar to the injected CML but could not be differentiated from a potential *de novo* CML. Difficulties in achieving CML disease in this model may be explained by the presence of minor antigen incompatibilities (miHAs), which may exist between host and tumor despite being MHC matched. Inbreeding can induce the loss (or gain) of expression of an immunogenic protein (secondary to a mutation) to which the animal may have not been made tolerant during thymic T cell education. Thus, host “rejection” of infused tumor cells can occur despite being MHC matched in the context of insufficient immunosuppression. This is supported by SQ tumor growth in the animal conditioned with high amounts of irradiation (500 cGy) while animals that received lower amounts of irradiation (100–300 cGy) did not exhibit any tumor growth. Two other explanations for failed tumor growth can be attributed to the loss of growth characteristics (growth factors, adhesion molecules, etc.) of the *in vitro* passaged CML tumor cells and the requirement of a longer time to develop *in vivo* than what was designed in the IACUC protocol.

To assess whether the *in vitro* culture process affected tumor cell growth capacity *in vivo*, cell lines were passaged *in vivo* in mice. Tumors have historically been expanded across xenogeneic barriers in immunodeficient mice (NOD/SCID) and have been successful in selecting for aggressive tumor subclones (Waller et al., 1993; Adam et al., 2007; Schook et al., 2015). Though not ideal to expand tumor cell lines in animals different from the original host species, this approach ensures that tumor cells retain their *in vivo* growth capacity. 14736 CML tumor cells did not grow in NOD/SCID mice (Cho et al., 2007), but did lead to CML disease in NSG (NOD/SCID gamma -/-) mice (Schenk et al., manuscript in preparation), albeit requiring over 4 months. These results suggest that the innate immune system of NOD/SCID mice may have been sufficient to “reject” the tumor cells, as NSG mice lack macrophages and NK cells. Thus, spontaneous swine CML lines can be successfully expanded *in vivo*, and transfer studies into swine are forthcoming.

## Porcine Lymphotropic Herpesvirus (Plhv) Induced B Cell Lymphomas

A major area of study in MGH miniature swine for the past 30 years has been the use of HCT to induce mixed hematopoietic chimerism without GVHD, both for solid organ tolerance and treatment strategies of hemolymphatic neoplasias. Currently, a major complication of both HCT and SOT is the development of post-transplant lymphoproliferative disease (PTLD). PTLD is observed in immunosuppressed transplant patients, but similar lymphoproliferative processes can present in those naturally immunosuppressed, such as AIDS patients (Bollard et al., 2004; Abu-Elmagd et al., 2009). Under the cover of immunosuppression and depressed CD8+ T cell immunity, the B cell population aggressively expands as a result of primary infection or reactivation of a herpes virus, most commonly Epstein Barr virus (EBV; Heslop et al., 1996; Lucas et al., 1996). Unfortunately, the variability of the human patient population, both clinically and pathologically, complicates the ability to study this disease (DiNardo and Tsai, 2010). Murine models of PTLD involving immunodeficient mice injected with human PTLD lines and mice infected with murine gamma herpesvirus are unreliable and do not accurately model human disease (Schiffman and Breen, 2015).

**FIGURE 1:**
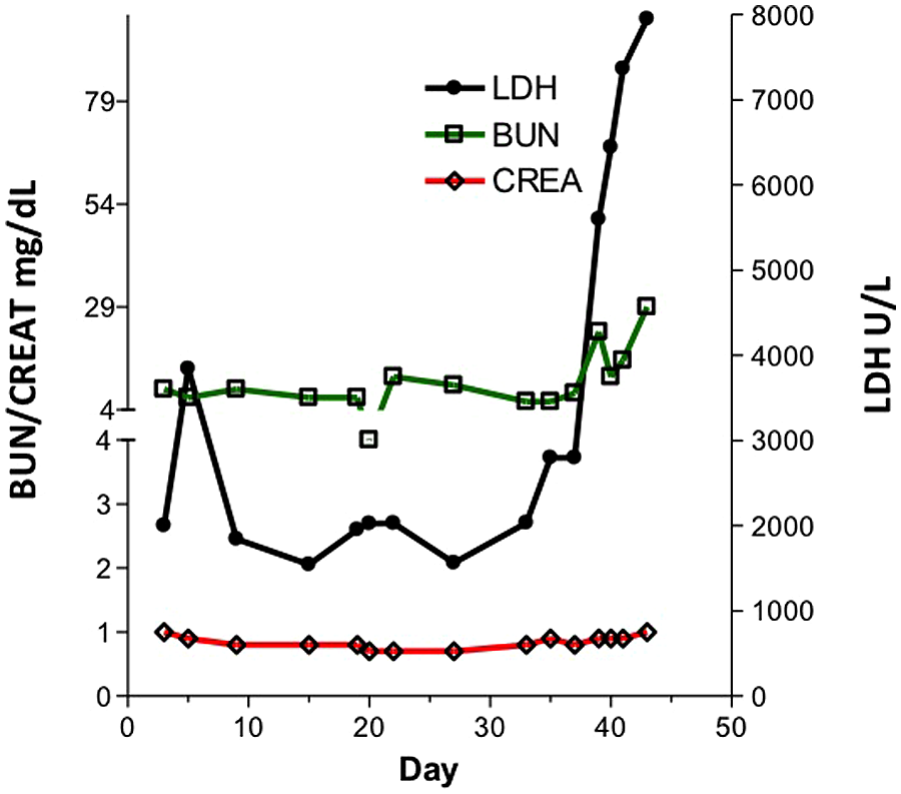
**LDH, BUN and Creatinine in a pig developing post-transplant lymphoproliferative disease (PTLD).** Kidney values remained relatively constant while LDH levels rose acutely.

**FIGURE 2:**
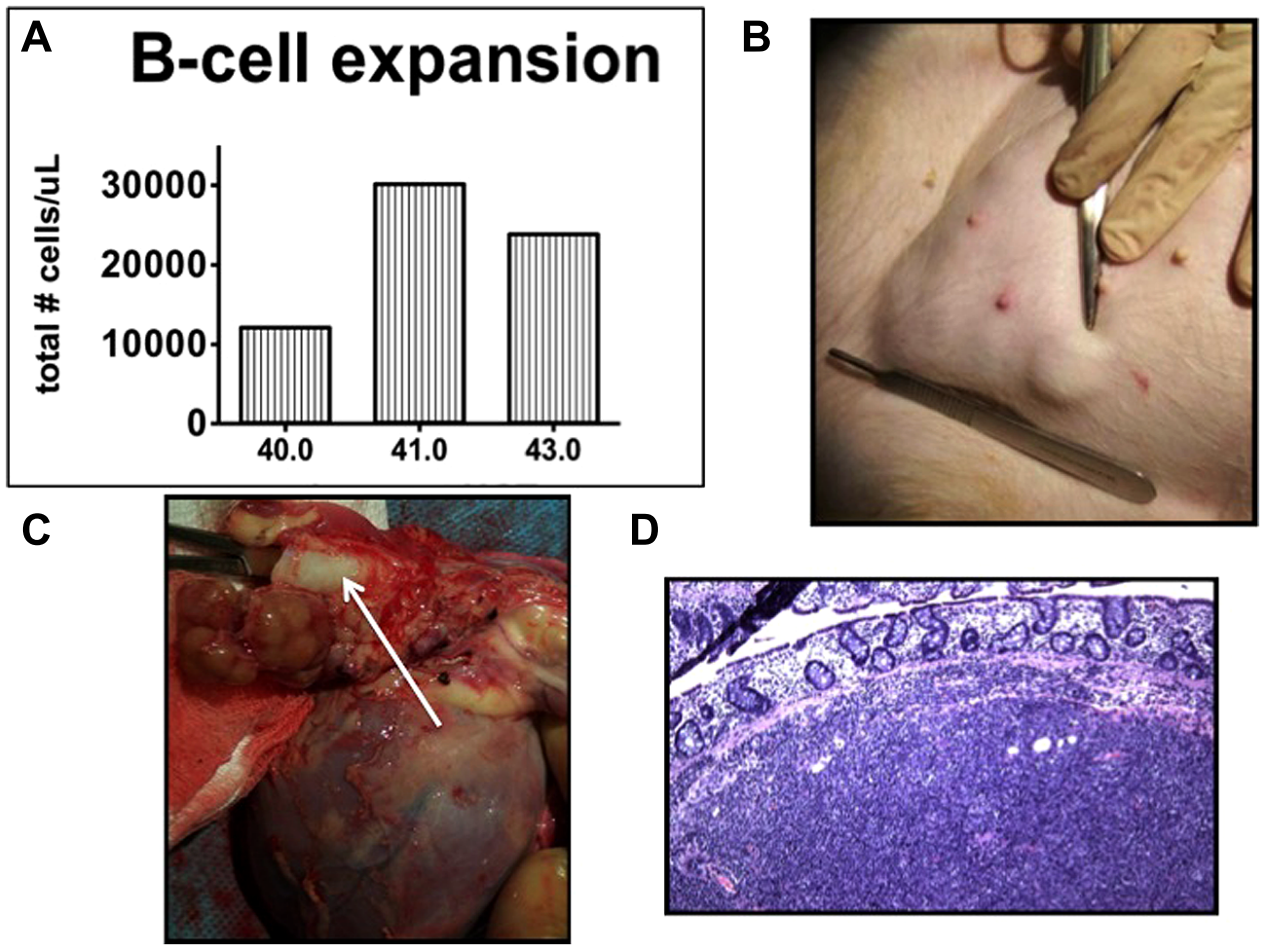
**Post-transplant lymphoproliferative disease in miniature swine.** **(A)** Total B cell burden in an animal developing PTLD. B cells tripled in number within 24 h. **(B)** Lymphadenopathy (inguinal area). **(C)** Perihilar lymphadenopathy (arrow shows trachea). Cranial aspect of the animal is to the left of the image. **(D)** H & E section demonstrating cellular infiltrate in a lymph node (20×).

In contrast, swine have been shown to be an excellent model for the study of PTLD. Immunosuppressed swine undergoing HCT or SOT develop B-cell expansions with a clinical presentation that closely resembles human PTLD (Huang et al., 2001; Matar et al., 2015). Similarly to human PTLD’s association with EBV, swine PTLD is associated with primary infection or reactivation of a gamma herpesvirus, porcine lymphotropic herpesvirus-1 (PLHV-1; Doucette et al., 2007). In a model of haploidentical HCT, immunosuppressive regimens consisting of T-cell depletion using CD3-immunotoxin, 1000 cGy of thymic irradiation, and a 30–60 days course of cyclosporine A consistently (40–50%) resulted in the development of B cell lymphomas post-transplant (Cho et al., 2004; Cina et al., 2006; Matar et al., 2015). When thymic irradiation was eliminated as part of the conditioning regimen, only 1/23 animals developed PTLD. However, in the absence of thymic irradiation, T cell depletion was poor, resulting in inconsistent stem cell engraftment. 100 cGy of TBI was added to the conditioning regimen in an attempt to decrease the incidence of PTLD while allowing for stem cell engraftment. Subsequently, only 15% developed PTLD, while the majority of animals successfully engrafted (Matar et al., 2015). Matar et al. (2015) recently explored the effect of thymic and TBI on the incidence of PTLD in this model and concluded that thymic irradiation was a risk factor for PTLD development via its depleting effect on the absolute number of T cells. Further, the use of LDH as a serum marker for swine PTLD was validated (**Figure 1**). As in humans, B cell expansion in the context of swine PTLD is mirrored by increases in LDH (Boothpur and Brennan, 2008), even before clinical signs of PTLD such as lymphadenopathy (**Figure 2**). This was also shown to be diagnostically valuable in swine CMLs (Duran-Struuck et al., 2010) which mirrored the LDH increases observed in human CMLs and reinforcing the swine tumor model.

In clinical practice, the standard treatment approach for PTLD is the reduction of immunosuppression and sometimes followed by a second treatment modality if necessary, such as rituximab (anti-CD20 mAb) or chemotherapy (DiNardo and Tsai, 2010). Removing immunosuppression in the transplant setting is often complicated by organ rejection or graft-versus-host disease in the setting of allogeneic bone marrow transplantation (BMT). Chemotherapy remains an effective, yet toxic treatment option, and rituximab though effective, does not control PTLD in all cases. In the context of swine PTLD following BMT, reduction or discontinuation of immunosuppression only sometimes leads to PTLD resolution, but GVHD is a common consequence (Duran-Struuck et al., manuscript in preparation). In the study cited above, of 11 animals that developed PTLD, only two cases resolved after discontinuation of cyclosporine, and those two animals subsequently developed GVHD. This naturally induced model of PTLD following BMT can be used to study novel treatment approaches such as new antivirals or the use of *in vitro* primed host CD8+ T cells as a cellular therapy for primary or refractory PTLD.

## Swine Ptld Tumor Lines

Although this naturally induced model offers a clinically relevant opportunity which to study PTLD, it is limited by the inconsistency in PTLD incidence (40–50%) and the logistics and cost involved in a BMT or SOT. Alternatively, swine PTLD tumors have been successfully harvested from various involved organs, including lymph nodes and spleen, and expanded *in vitro* with the intention of establishing an immortal cell line that can reliably induce PTLD when introduced into a naïve animal from the same inbred line. To test the ability of these cell lines to grow *in vivo*, as a preliminary experiment, a PTLD B cell lymphoma line was established from animal 13271 and infused intravenously into unconditioned NSG (NOD/SCID IL-2r gamma-/-) mice (Schenk et al., manuscript in preparation). In general, an average of 10 × 10^6^ PTLD cells was infused per mouse. At this dose, we observed successful “engraftment” of tumor cells with 100% lethality within 57–70 days. Subsequently, the same PTLD cell line was tested in two MHC matched swine. Animals were preconditioned with a non-myeloablative protocol that has previously been permissive for the induction of PTLD. Animals received 100 cGy of TBI on day -2, T cell depletion with a recombinant CD3-immunotoxin twice daily from day -4 to day -1, and were maintained under cyclosporine coverage for 60 days. In total, each animal received three doses of approximately 300 × 10^6^ tumor cells/kg over a period of 1 week, totaling 900 × 10^6^ tumor cells/animal. Tumor cells were infused intravenously (IV) and/or intraosseously (IO), with the intention of overwhelming the animals’ tumor clearing capacity and allowing for successful “engraftment” of tumor cells. Unfortunately, none of the animals developed PTLD. B cell counts normalized soon after infusion and an increase in B cells was only observed during the peri-infusion period as determined by flow cytometry analysis. Thus, PTLD tumor cells selected *in vitro* for their growth ability and which had successfully engrafted in NSG mice did not cause overt PTLD in immunocompromised miniature swine. A limitation of this non-myeloablative approach is the potential for radiation resistant T cells to “reject” the tumors via minor antigen incompatibility (Nadazdin et al., 2011; Zhang et al., 2012) or TLR ligation (Yu et al., 2012). Thus, conditioning regimens that have a stronger depleting effect on T cells, such as the use of thymic irradiation which has been shown to be very conducive to the development of PTLD, may be required to better induce tumor growth.

From an immunologic standpoint, it is crucial to understand the method of “graft” (tumor) loss in this model. There are several possibilities for the lack of tumor cell engraftment including an active rejection of tumor cells by residual host defenses, evasion of host immune responses by “hiding” in an immune privileged site (such as the bone marrow), simply being ignored by host defenses in the circulation, or alternatively, tumor cells may have died due to lack of fitness, without an immunological attack. In our studies, mixed lymphocyte reactions (MLR) and cell mediated cytolysis (CML) assays did not suggest a cellular sensitization against tumor antigen(s). This implies the mechanism of graft loss was possibly non-immunologic, either via clearance from the circulation or lack of fitness in the swine environment.

Due to the fact that host and tumor cells are fully MHC matched, it is difficult to distinguish tumor cells after infusion, as they could be residing in the marrow or lymph nodes without surviving in the peripheral blood. As a method to distinguish and monitor tumor cells *in vivo*, a green fluorescent protein (GFP) gene was transduced into the PTLD tumor cell line using a lentivirus vector. GFP(+) tumor cells were then sorted and expanded. Interestingly, GFP+ tumor cells grew faster *in vitro* compared to GFP(-) tumor cells. When NSG mice were challenged with GFP(+) tumor cells, we observed a faster onset of disease as well as more extensive organ involvement, suggesting a more aggressive tumor. One MHC matched swine was infused with GFP(+) PTLD under the same conditioning regimen as used previously. The cells were monitored via flow cytometry and were undetectable after 48 h, suggesting they were either cleared from the circulation or sequestered. Again, no sensitization was observed by MLR or CML assays indicating a non-immunologic mechanism of graft loss (Schenk et al., manuscript in preparation).

## Conclusion And Future Directions

Spontaneously arising hemolymphatic tumors in the MGH miniature swine herd, specifically myeloid leukemias and B cell lymphomas, closely resemble human malignancies, making the MGH swine an valuable model for the development of a clinically applicable large animal tumor model based on their unique genetic characteristics. Future approaches focusing on reproducibility will include several strategies, including; (i) optimizing transplant protocols to induce tumor cell engraftment, (ii) *ex-vivo* transduction of porcine hematopoietic stem cells with known oncogenes (Adam et al., 2007), and (iii) the introduction of oncogenes via retroviral vectors (Adam et al., 2007). Alternatively, backcrossing the MHC characterized mini-swine with the first naturally occurring severe combined immunodeficient (SCID) pig line. This SCID pig has already been shown to accept human tumor xenografts, and thus can enhance the engraftment of allogeneic tumor transfer studies (Waide et al., 2015). In summary, the importance of a consistently reproducible large animal tumor model cannot be understated, as it will facilitate the study of these lethal malignancies and test reliably novel therapeutic strategies for clinical applications.

## Conflict of Interest Statement

The authors declare that the research was conducted in the absence of any commercial or financial relationships that could be construed as a potential conflict of interest.
